# Clinically assessed consequences of workplace physical violence

**DOI:** 10.1007/s00420-014-0950-9

**Published:** 2014-06-15

**Authors:** Jacqueline De Puy, Nathalie Romain-Glassey, Melody Gut, Wild Pascal, Patrice Mangin, Brigitta Danuser

**Affiliations:** 1 Centre universitaire romand de médecine légale, Lausanne, Switzerland; 2 Institut national de recherche et de sécurité pour la prévention des accidents du travail et des maladies professionnelles, Nancy, France; 3 Institut universitaire romand de santé au travail, Lausanne, Switzerland

**Keywords:** Physical assault, Psychological condition, Organizational support, Longitudinal study, Predictors, Severity score, Consequences of violence

## Abstract

**Objectives:**

To assess consequences of physical violence at work and identify their predictors.

**Methods:**

Among the patients in a medicolegal consultation from 2007 to 2010, the subsample of workplace violence victims (*n* = 185) was identified and contacted again in average 30 months after the assault. Eighty-six victims (47 %) participated. Ordinal logistic regression analyses assessed the effect of 9 potential risk factors on physical, psychological and work consequences summarized in a severity score (0–9).

**Results:**

Severity score distribution was as follows: 4+: 14 %; 1–3: 42 %; and 0: 44 %. Initial psychological distress resulting from the violence was a strong predictor (*p* < 0.001) of the severity score both on work and long-term psychological consequences. Gender and age did not reach significant levels in multivariable analyses even though female victims had overall more severe consequences. Unexpectedly, only among workers whose jobs implied high awareness of the risk of violence, first-time violence was associated with long-term psychological and physical consequences (*p* = 0.004). Among the factors assessed at follow-up, perceived lack of employers’ support or absence of employer was associated with higher values on the severity score. The seven other assessed factors (initial physical injuries; previous experience of violence; preexisting health problems; working alone; internal violence; lack of support from colleagues; and lack of support from family or friends) were not significantly associated with the severity score.

**Conclusions:**

Being a victim of workplace violence can result in long-term consequences on health and employment, their severity increases with the seriousness of initial psychological distress. Support from the employer can help prevent negative outcomes.

## Introduction

There has been in recent years a growing awareness and media coverage about psychological harassment at work and its devastating impact on victims, such as stress or burnout syndromes (Tarquinio et al. 2004) (Bowling and Beehr 2006; Hansen et al. 2006). Physical forms of workplace violence have been investigated as well, but there has been comparatively little research on consequences of physical assaults against workers. As a matter of fact, many studies and reviews have concentrated on identifying risk factors and assessing the prevalence of this phenomenon (Barling et al. 2009; Dillon 2012). The healthcare setting has drawn particular attention (Gillespie et al. 2010; Kowalenko et al. 2012; Taylor and Rew 2011). Acts of physical violence at work are defined as assaults carried out by one or several perpetrators, by members of the same organization as the victim (internal violence) or by “outsiders” (external violence) such as clients and patients. External forms of physical violence are more common than internal ones and affect more often, but not exclusively, “frontline staff” in the services industry (European Foundation for the Improvement of Living and Working Conditions 2007). Workplace violence seems to become more pervasive throughout the world and represents a growing health and security challenge for many organizations. An increase in the prevalence of physical workplace violence (from 4 to 6 % in the past 12 months) was reported in the European Working Conditions Surveys from 1995 to 2005 in Northern Europe. The same study showed that external physical violence was more frequent than internal physical violence. Substantial differences were observed according to the type of occupation. The highest rates were found in the health and social work sectors (15 %), public transportation (12 %), public administration (11 %), hotel or restaurants (8 %), and education (8 %) (European Foundation for the Improvement of Living and Working Conditions 2007; Graf et al. 2007). A Swiss study investigated frontline staff in Switzerland from regional services for placement of the unemployed and showed that 21 % of the respondents reported physical violence from clients (Mueller and Tschan 2011). As far as gender and age are concerned, there are contradictory findings across studies. Differences may be partly due to the fact that they concern different countries or they may be caused by variations in methodologies. The European Working Conditions Survey did not reveal any differences between men and women in risks of victimization. However, in Great Britain, the British Crime Survey (Buckley et al. 2010) as well as a longitudinal study (Sprigg et al. 2010) found that men were more often assaulted at work than women. A Danish study (Wieclaw et al. 2006) indicated that women were three times more at risk of workplace violence than men. According to the British Crime Survey (Buckley et al. 2010), there was an interaction between age and gender. Among those aged 35–44, the prevalence of workplace violence was high and identical for men and women. Among those aged 25–34, men were more often the victims, while women aged 50 and more were more often the victims. A vulnerability of women over 50 was also found at the European level in the ECWS (European Foundation for the Improvement of Living and Working Conditions 2010).

Physical workplace violence has been shown to carry health consequences for victims, to affect the morale of teams and organizations, and to generate economic costs for employers, health and social services (Hogh and Viitasara 2005; Tarquinio et al. 2004; Wieclaw et al. 2006). A lack of methodological and conceptual consistency across studies in this field and a shortage of longitudinal designs have been pointed out (Sprigg et al. 2010). Consequently, there is still limited evidence on consequences of physical workplace violence and how they may impact victims differently according to their gender and age.

The aim of the present research project was to investigate physical workplace violence and its consequences in a clinical sample of victims consulting a violence medicolegal unit in the regional university hospital in Lausanne, Switzerland. The objectives of the Violence Medical Unit (VMU) are twofold. First, the unit provides medicolegal consultations to victims of interpersonal violence. Second, the unit conducts research and teaching activities focused on the experience of victims of violence and the responses of professionals who provide care. Under the supervision of forensic pathologists, nurses independently provide consultations to victims of violence. Typically, a consultation lasts about 2 h. It starts with attentive listening and debriefing of the patient, followed by a clinical examination which includes photographs of wounds, and is concluded by evaluating the victim’s needs and providing advice on where to find additional help and support. The VMU produces a battery and assault report that can be used to support the filing of a complaint. Since the unit opened in 2006, the number of consultations has steadily increased from 529 in 2006 to 891 in 2013. On average, 30 % of the victims consulting the VMU indicated they were subjected to physical domestic or family violence and 70 % declared being victims of a physical violence assault that took place in the community (Romain-Glassey et al. 2009).

The present project was developed and carried out in collaboration with the Institute of Health at Work and focused on workplace violence victims in Switzerland. An interdisciplinary team of specialists in occupational health and in violence prevention (medical doctors, nurses, social scientists and a biostatistician) collaborated in all stages of the study. The research questions were defined as follows: (1) among the population of patients who sought assistance from the unit between 2007 and 2010,^1^ how many were workplace violence victims? (2) What were the socio-demographic characteristics and occupations of workplace violence victims and what were the characteristics of the violent events? (3) What were the clinically assessed consequences of these events on the health and work of the victims and what factors increased the severity of consequences?

## Methods

### Study design

The research protocol for the present study was approved by the regional Ethics Committee on Human Experimentation on February 1, 2011, in accordance with the Helsinki Declaration (World Medical Association 2000). Participants in the study were identified and selected by screening all medicolegal files (*N* = 1,257) concerning events of community violence reported by patients of the VMU medicolegal consultation in the Lausanne University Hospital between January 1, 2007, and December 31, 2010. During a consultation, the attending health professional takes extensive notes and fills in a patient’s file with questions grouped in six sections (see Appendix 1).

The source population of workplace violence victims was composed of 185 patients who reported 196 violent events. Nine patients experienced multiple (2–3) occurrences during the 4-year period considered.

During the follow-up study carried out in the summer of 2011, it was planned to reach all 185 patients who had given their consent to be contacted again. However, two did not have a phone number, and nine did not speak French or another language spoken by the two interviewers. Eighty-three persons could not be found, either because the phone number was no longer valid or because there was no reply after at least eight attempts at different times of the day and evening, on two different weekdays. Eighty-seven respondents agreed to participate, and 15 did not give their consent. The subjects were informed on the nature of the study and were explained that they could refuse to participate, interrupt the interview or not answer any question at their convenience. One interview was considered invalid, because it was conducted with the victim’s husband. Among the 86 victims who participated in the follow-up study, two had consulted for three different events of violence and three for two events. These five persons were interviewed about the most recent event.

### Measures

The variables listed below were taken into account and were based on the information contained in the medical files. Given the small size of the sample, values were grouped in a maximum of 3–4 categories, with the exception of the occupational classification variable.

*Socio*-*demographics*: age (<35/35–44/45+), gender, nationality (Swiss/non-Swiss); foreigners with a work and residence permit (yes/no); and highest level of education (compulsory or no school/vocational education and training/high school and beyond).

*Work situation:* type of occupation (14 categories); occupational status (employee/self-employed); and occupational sector (agriculture/industry/services).

*Medical history:* generally in good health (yes/no); and previous experience of violence (yes/no).

*Characteristics of the violent event*: type of workplace violence (internal/external/both internal and external); internal violence perpetrator (subordinate/colleague/superior); and time of the assault (day work: 7 a.m. to 7 p.m./evening work 8–10 p.m./night work 11 p.m. to 6 a.m.).

A measure to categorize occupations according to the degree of organizational and personal awareness as well as risk of workplace violence (low/moderate/high) was developed in the qualitative section of the study as a result of a thematic content analyses of the respondents’ statements (De Puy et al. 2012). These three degrees of awareness were also characterized by different grades of surprise and shock at being assaulted at work.

The “high risk and awareness of violence jobs” category included occupations where the risk of violence was systematically considered as “part of the job” by respondents (police officers, prison guards, private security agents and public transportation ticket controllers). These job holders explained that they were prepared and trained to meet aggressive resistance when controlling, arresting or sanctioning subjects. They mentioned that their organizations had protocols for dealing with such events. In these “high risk and awareness of violence jobs,” assaults were never deemed normal but they were considered by respondents as a frequent and expected occupational risk.

The “moderate risk and awareness of violence jobs” category included occupations in contact with the public on a daily basis (taxi drivers, bus drivers, salespersons, post office staff, healthcare staff, social workers, waiters, teachers, janitors and sex workers). Those who held “moderate risk and awareness of violence jobs” provided different types of services to customers, patients, etc. and were aware of the risks of violence under certain circumstances. These circumstances included threats and acts of violence by angry and/or inebriated persons, or perpetrators of thefts and holdups. Among workers holding “moderate risk and awareness of violence jobs,” the element of surprise and shock after an assault was present but respondents were aware of similar events and perceived growing risks in their profession which they often attributed to societal trends (e.g., loss of respect for their profession, increase in crime, verbal abuse or violence).

Workers who had no regular contact with the public were included in the “low risk and awareness of violence jobs” category (administrative personnel, blue collar workers, farm workers and kitchen staff). When these types of workers were faced with physical violence, they described the violence as surprising and unexpected (for instance, a lorry driver who was assaulted when delivering goods or a clerk who was attacked by a colleague during a company dinner).

### Predictor variables

Based on the clinicians’ experience and on risk factors identified in previous studies, we selected six predictors (collected during the medicolegal consultation) and three risk factors^2^ (reported during the follow-up interviews). Each predictor and risk factor was deemed likely to influence negative consequences on the victim’s health and work. Predictors were (a) clinically assessed symptoms of psychological distress resulting from the violent event; (b) clinically assessed physical wounds resulting from the violent event; (c) internal violence vs. external violence; (d) generally not in good health (i.e., preexisting health problems); (e) previous experience of violence; and (f) working alone when assaulted. Considered risk factors were as follows: (1) perceived lack of support from the employer; (2) perceived lack of support from colleagues; and (3) perceived lack of support from family and friends. Variables were dichotomized with a zero value in the absence of the measured factor and a value of 1 in its presence, except for initial physical wounds and psychological distress which were given four values: 0 (none), 1 (minor), 2 (moderate) and 3 (severe).

### Outcome variables

An innovative method of scoring and assessing clinically the severity of health and work consequences of violent events was constructed by a panel of experts from the Institute of Health at Work and the University Center for Legal Medicine. It was agreed to add the values of three variables: (V1) physical health consequences; (V2) psychological health consequences; and (V3) negative consequences on work. The values for these variables were attributed according to the severity of each consequence: 0 (no consequences); 1 (minor consequences); 2 (moderate consequences); and 3 (severe consequences). Examples are provided in Appendix 2. Values for physical and psychological consequences were attributed and cross-validated for each case by the three medical doctors in our team. They clinically evaluated each complaint according to whether it constituted a minor, moderate or severe hindrance in performing daily activities. Theoretical values of the severity score range from 0 (none of the measured consequences) to 9 (maximum severity).

### Statistical analysis

By means of ordinal logistic regression analyses (proportional odds), each predictor was included separately as an independent variable for a priori selection of factors. Then, all identified factors were introduced jointly. Finally, a backward stepwise selection was applied. The dependent variable was the severity index. However, since the Cronbach alpha value for the score was found to be low (0.51), separate multiple stepwise regression analyses with each component of the score as the dependent variable were performed as well (consequences on work; psychological consequences; and physical consequences) using the list of independent variables selected for the global severity index. Coefficients were exponentiated. One possible interpretation of these exponentiated coefficients of the ordinal logistic regression is that they are odds ratios at any arbitrary cut point of the ordinal outcome variable. Statistical analyses were performed with Stata_/IC 11.1 (StataCorp_ 2009 LP). Gender and age were introduced as covariates.

## Results

Our first two research questions aimed at identifying the characteristics of patients who had been victims of workplace violence and the characteristics of the workplace violence events that had motivated them to consult. Answers to these questions were provided by means of descriptive statistics. Table 1, Appendix 4 and 5 present these results in detail.

**Table 1:** Comparative statistics of baseline and follow-up population, by gender

Variables	Baseline population *N* = 185				Follow-up population *N* = 86			
	Male *N* = 129		Female *N* = 56		Male *N* = 67		Female *N* = 19	
Mean age (SD)	39	(12)	37	(11)	40	(12)	42	(12)
Age-groups	*N*	%	*N*	%	*N*	%	*N*	%
<35	54	42	27	48	25	37	5	26
35–44	35	27	15	27	20	30	6	32
45+	40	31	14	25	22	33	8	42
Interviewed <12 months after the consultation								
No					57	85	14	74
Yes					10	15	5	26
Degree of risk and awareness of workplace violence and type of occupation								
High risk and awareness of violence	46	36	4	7	26	39	–	
Private security agents	26	20	1	2	13	19	–	
Police officers/prison guards	12	9	2	4	7	11	–	
Ticket inspectors (public transportation)	8	6	1	2	6	9	–	
Moderate risk and awareness of violence	51	40	39	70	27	40	16	84
Taxi drivers	12	9			7	11	–	
Salespersons, retail business owners	11	8	7	12	5	7	2	10
Service staff in hotels, restaurants, bars/discos	10	8	10	18	5	7	1	5
Health, teachers, social workers, school librarian	6	5	14	25	3	4	11	58
Drivers (public transportation)	5	4	–		4	6	–	
Sex workers	1	1	6	11	–		2	10
Janitors	4	3	2	4	2	3	–	
Post office staff (counter)	2	2	–		1	2	–	
Low risk and awareness of violence	32	24	13	23	14	20	3	16
Administration	7	5	7	13	3	4	2	11
Misc. blue collar (construction and factory workers, auto-mechanics, truck drivers, etc.)	22	17	5	9	10	15	1	5
Kitchen staff	3	2	1	2	1	1.5	–	
Highest level of education								
Compulsory or no school	30	23	18	32	17	25	4	21
Vocational education and training	46	36	8	14	24	36	3	16
High school and beyond	28	22	15	27	18	27	8	42
Missing values	25	19	15	27	8	12	4	21

### Characteristics of the workplace violence victims

Since it was deemed important to examine differences between men and women, tables were broken down by gender. In brief, we found that the total population of workplace violence victims was composed of 185 patients who reported 196 violent events. Seventy percent of the victims were male. The youngest age-group (under 35) was the most represented category, both for men (42 %) and women (48 %). Ninety-two percent of respondents worked in the service industry and in contact with the public. Among the types of occupations held by the victims, 36 % of men worked in “high risk and awareness of violence jobs” (private security agents, police officers, prison guards and ticket controllers in public transportation), while only 7 % of the women were found in that category. Seventy percent of women vs. 40 % of men were employed in “moderate risk and awareness of violence jobs.”

### Characteristics of the workplace violence events

Concerning characteristics of the violent events (*N* = 196), 73 % of situations concerned external violence and 27 % internal violence. The latter were perpetrated in 70 % of cases by a colleague, 24 % of the time by a subordinate and more rarely (6 %) by a superior. The perpetrator acted alone in 83 % of situations, and 91 % of the time was male. Thirty-two percent of the violent events happened during night work (11 pm–6 am). In all cases, victims were assaulted physically.

### Consequences of the workplace violence events

Our third research question aimed at investigating the clinically assessed consequences of the workplace violence events on the health and work of the victims, and at identifying factors that affected the severity of consequences. To this end, a follow-up study was carried out.

Table 1 allows comparison of the source population with the population of patients who participated in the follow-up telephone survey (*N* = 86). The two most noteworthy differences between the baseline and source population were, first, a higher male/female sex ratio (3.5) and, second, a larger representation of Swiss citizens (55 %) than foreign nationals (45 %). As far as the other variables examined were concerned, the two populations were quite similar. Telephone interviews were carried out between 7 and 55 months after the violent event, with an average of 30 months.

The severity of consequences of the workplace violence event was scored. The maximum severity score value recorded was 7/9. Fourteen percent scored ≥4, which corresponds to particularly severe consequences. Forty-two percent were in the medium range of the score (1–3). For 44 % of interviewees, scores were zero in the absence of consequences. Values were significantly higher for women than for men (*p* = 0.02). Although values increased with age, this trend was no longer significant when taking into account gender. Table 2 shows consequences of the workplace event (components of the severity score) by gender.

**Table 2:** Consequences of the workplace violence event

	Follow-up population (*N* = 86)			
	Males (*N* = 67)		Females (*N* = 19)	
Type of consequence	*N*	%	*N*	%
Initial symptoms of psychological distress				
None	29	43	2	11
Minor	20	30	4	21
Moderate	14	21	8	42
Severe	4	6	5	26
Perception of the employer’s response				
Adequate	33	50	6	31
No employer	10	15	3	16
Inadequate	23	35	10	53
Missing value	1	2	–	–
Previous experience of violence and jobs with high risk and awareness of violence				
No/other jobs	29	43	11	58
No/high risk and awareness of violence jobs	6	9	–	–
Yes/other jobs	11	16	8	42
Yes/high risk and awareness of violence jobs	20	30	–	–
Missing value	1	2	–	–
Psychological consequences				
None	37	55	10	53
Minor	21	31	–	–
Moderate	5	7	5	26
Severe	3	5	4	21
Missing value	1	2	–	–
Physical consequences				
None	52	78	12	63
Minor	14	21	7	37
Moderate	1	1	–	–
Severe	–	–	–	–
Adverse effect on work and employment				
None	34	50	4	21
Sickness leave but no lasting effect on job	24	36	7	37
Diminished work time	1	2	1	5
Left the job or was dismissed	8	12	7	37
Severity score values				
0	19	28	2	11
1–3	38	58	11	58
4+	9	14	6	32
Missing value	1	–	–	–

Among potential predictors of severity considered, only sex, age classes, previous violence victimization, initial symptoms of psychological distress, and jobs with high risk and awareness of violence were statistically significant when tested alone. Therefore, these predictors were further considered in the analyses. In view of the large variation in follow-up times, we tested through a regression analysis whether the time elapsed (in months) since the consultation and the follow-up interviews had any effect on the severity score. For instance, it could be expected that the most recent violent events would be associated with higher values of the severity score. However, no such effect was observed.

The following four variables were not associated with the severity score in a statistically significant way: internal vs. external violence; pre-existing health problems; working alone at the time of event; and initial physical wounds. Moreover, two variables (previous experience of violence; and jobs with high risk and awareness of violence) were negatively related to severity and positively correlated. Consequently, we tested the interaction between these two variables and found that the results for prior violent victimization were very different for jobs with high risk and awareness of violence. Consequently, we included the interaction of these two variables. Among the risk factors assessed during the follow-up interview, namely perceived support from family and friends, perceived support from colleagues, and perceived support from the employer, only the latter, i.e., absence or lack of support from the employer, was significantly related to severity. The cross-tabulation of all variables with the severity score regrouped in three categories is given in Appendix 5.

Table 3 presents the odds ratios of the full model including all the variables selected in the above step as well as the model which is the result of the backward selection with a 5 % *p* value for removal. All variables with several categories (e.g., age classes) were either removed or kept jointly.

**Table 3:** Ordinal logistic regression analyses of predictors on the severity score

	Full model^a^		Selected model^b^	
	OR	95 % CI	OR	95 % CI
Gender				
Male	–			
Female	2.20	0.73, 6.61		
Age				
<35				
35–44	0.74	0.25, 2.17		
45 and more	1.13	0.38, 3.39		
Initial symptoms of psychological distress				
None	–		–	
Minor	3.25	1.03, 3.43	3.02	0.99, 9.23
Moderate	4.80	1.40, 16.5	5.47	1.71, 17.5
Severe	44.4	7.95–248	54.2	10.7, 275
Perception of the employer’s response				
Adequate	–			
No employer	3.90	1.12, 13.5	3.73	1.09, 12.8
Inadequate	2.87	1.04, 7.94	2.86	1.06, 7.66
Previous experience of violence and jobs with high risk and awareness of violence				
No/other jobs	–		–	
No/high risk and awareness of violence jobs	13.0	2.43, 69.9	11.0	2.08, 58.3
Yes/other jobs	0.54	0.18, 1.63	0.70	0.25, 1.97
Yes/high risk and awareness of violence jobs	0.72	0.22, 2.37	0.61	0.19, 1.90

^a^Model including jointly all factors which were statistically significant in simple regression analyses

^b^Model obtained from the full model by backward selection

The strongest feature of the regression analysis was that the severity score increased with the severity of the initial symptoms of psychological distress. On the other hand, age and sex were no longer found to be significant independent variables. The analysis of the interaction between previous experience of violence and “high risk and awareness of violence jobs” vs. “other jobs” (i.e., “moderate and low risk and awareness of violence jobs”) revealed notable results. First, in the “other jobs,” previous experience of violence did not affect severity of consequences of the violent event. Second, in the “high risk and awareness of violence jobs,” the severity score was higher in the group without previous experience of violence.

The significance of independent variables differed when considering their effect on the three components of the severity score taken separately (Table 4). For psychological consequences, the significant independent variables were initial symptoms of psychological distress and perceived lack of support from employer. For the consequences on work and employment, only severe initial symptoms of psychological distress were significant. For physical consequences of violence, only “no employer” (i.e., being an independent worker) was significant.

**Table 4:** Ordinal logistic regressions of independent variables on components of the severity score

	Consequences on work		Psychological consequences		Physical consequences	
	OR	95 % CI	OR	95 % CI	OR	95 % CI
Initial symptoms of psychological distress						
None	–		–			
Minor	1.4	0.50–4.17	4.97	1.32–17.7		
Moderate	3.3	1.13–9.73	3.29	0.80–13.5		
Severe	19.7	4.34–89.6	30.475	5.14–180.2		
Perception of the employer’s response						
Adequate			–		–	
No employer			7.04	1.73–28.7	8.12	1.62–40.7
Inadequate			3.88	1.21–12.4	2.53	0.66–9.69
Previous experience of violence and job with high risk and awareness of violence						
No/other jobs			–			
No/high risk and awareness of violence jobs			8.30	1.43–48.1	8.49	1.28–56.3
Yes/other jobs			0.68	0.21–2.24	0.62	0.16–2.42
Yes/high risk and awareness of violence jobs			0.88	0.20–3.90	0.55	0.10–3.20

## Discussion

We found a strong association, in a multivariable model controlling for gender, between signs of initial psychological distress and the severity of consequences several months after a workplace violence event. Although we did not find a direct effect of gender in the multiple regression analyses, initial symptoms of psychological distress were more prevalent and severe for women than for men. Moreover, among victims in high violence risk and awareness of violence occupations, more severe consequences were recorded for those who had no prior experience of violence. We also found that a perceived lack of support from the employer tended to increase the severity of consequences.

Our results are consistent with previous studies in other countries which have indicated that psychological consequences of workplace violence can be serious (Hogh and Viitasara 2005; Tarquinio et al. 2004; Wieclaw et al. 2006). Our findings are also comparable to those from a study by Mueller and Tschan (2011) which showed that the experience of workplace violence resulted in fear of violence, impaired psychological and physical wellbeing, and irritability. Similarly, Rogers and Kelloway (1997) found that fear of future violence following exposure to occupational violence predicted psychological well-being, somatic symptoms and intent to leave the organization. However, in light of our qualitative study results (De Puy et al. 2012), the severity of the consequences of workplace violence seem to be explained by a broader set of circumstances than fear of future violence. Our qualitative results indicate that unresolved financial and psychological sequels of the past violent event seem sometimes to weigh more on the victims than the fear of future violence. For instance, several of our respondents reported important financial constraints associated with the loss of their job because of the violent event. Others, although they had retired or made a transition to a job with less exposure to violence, reported lasting psychological conditions that suggest post-traumatic stress disorders or depression.

Contrary to some previous research (LeBlanc and Kelloway 2002), we did not find evidence that internal workplace violence resulted in more negative outcomes than external violence. It is of interest to compare our findings regarding the role of employer support in reducing the seriousness of workplace violence consequences with those found by Schat and Kelloway (2003). Their study, carried out in a healthcare setting, demonstrated that organizational support moderated the effects of physical violence, vicariously experienced violence, psychological assault on emotional well-being, somatic health and job-related affect. Cole et al. (1997) showed that reduced supervisory support was associated with harassment, threats and fear of violence in the workplace. Our study points to the fact that employer support of employees is likely to be crucial to their recovery from a workplace violence event in a large variety of professions. Past research has often concentrated on one type of occupation, for instance in the healthcare sector (Gates 2004).

Our study has implications for the prevention of consequences of workplace violence by such interested parties as employers, occupational health and healthcare providers as well as victims’ services organizations. Based on our findings, the psychological distress of victims shortly after a violent event, even in the absence of serious physical injuries, should not be underestimated and victims should be advised to seek professional help. Moreover, the importance of support from employers for the recovery of workplace violence victims needs to be emphasized.

In the qualitative section of our study (De Puy et al. 2012), respondents gave examples of forms of support from employers that had been particularly helpful. This included moral support and follow-up (a phone call, a letter, or a visit to the hospital), assisting the victim in order to obtain medical care, legal and administrative advice (filing a complaint, or getting insurance benefits), and organizational measures to prevent future incidents (hiring security guards, improving protective procedures, banning the perpetrator from the premises or signaling the perpetrator to the staff). In contrast, interviewees who had not received any of these forms of support or had experienced the employer’s response as inadequate (e.g., victim blaming, being dismissed) expressed strong feelings of disappointment and distress.

We found that first-time victimization appears as a risk factor for more severe consequences in occupations with high risk and awareness of violence. This unexpected result would need to be verified in further studies with larger samples. However, it is possible that successful recovery and subsequent return to work after the violent encounter is the key factor rather than the number of times a violent incident is experienced.

The limitations of our study are inherent to the clinical nature of our population. The size of our sample was determined by the number of people who came to the consultation between 2007 and 2010 following a workplace violence event. It is likely that this represented only a portion of all victims of this type of aggression in the region. The victims in our sample were those who chose to consult with the unit for advice and assistance as well as to document the violence in a manner than could be used to support legal process. Most victims came through the emergency room of the hospital after receiving medical care. This population therefore could represent the “tip of the iceberg” of the most serious situations, i.e., those that required medical attention. Besides, people who seek medical attention in private practice are not systematically referred to the Violence Medical Unit. Our relative small sample size limits the power of the statistical findings which should also be viewed in relation to a possible type I error given the number of tests performed. Finally, although we did not notice significant statistical differences based on socio-demographic characteristics between the source population and the respondents to the telephone survey, we note that approximately half of the workplace violence victims could not be reached for follow-up.

In conclusion, we believe our study shows the relevance and need for further research on workplace violence victims, especially through longitudinal designs and a combination of quantitative and qualitative methods. There is a need to verify in larger samples the initial psychological impact on victims of workplace violence, especially in a variety of occupations. Furthermore, the moderating effect of employer support deserves further investigation. Our findings suggest the need for employer responsiveness and policies to reduce the impact and costs of workplace violence for society, organizations and victims.

## Appendix 1: The six sections of the patient’s file

1. General data: gender, age, contact information (address, phone numbers), family doctor
2. Socio-demographic data: nationality, marital status, education level and occupation
3. Data concerning the violent event that motivated the consultation: date, time and place. Information on the perpetrator(s): number, gender, known/unknown by the victim; nature of the assaults (physical, sexual, psychological violence, deprivation or neglect), threats, complaint filed or intention to do so.
4. Data concerning the clinical examination centered on the experience of violence : including number of medical consultations related to the violent event, previous violence victimization, situation and nature of wounds.
5. Data concerning complementary examinations.
6. Conclusions, assault and battery report established at the end of the consultation.

## Appendix 2

See Table 5.

**Table 5:** Variables and values of clinically assessed consequences of the workplace violence event, with examples

Clinically assessed physical consequences	
None = 0	Respondent indicates having fully recovered physically from the assault
Minor = 1	Examples:
	minor scars with no functional impairment nor significant disfigurement
	occasional headaches or muscular-joint pain alleviated by simple antalgic drugs
	discomfort after a nose fracture (feeling the nose is obstructed)
Moderate = 2	Examples:
	discomfort when eating, consecutive to the loss of teeth (was hit in the jaw) and consecutive use of a denture
Severe = 3	None recorded
Clinically assessed psychological consequences	
None = 0	Respondent indicates having fully recovered psychologically from the assault
Minor = 1	Examples:
	some amount of mistrust and bitterness,
	feels slightly anxious, sometimes thinks about the assault
	was clinically depressed but recovered
	keeps a low profile but finds it difficult and frustrating
	feels bitter and resentful
	is worried and suspicious. Avoids risky locations
	resumed smoking
Moderate = 2	Examples:
	very suspicious and vigilant
	has conducts of avoidance such as refusing to go to certain neighborhoods
	partially overcame the consequences of the violent event; finds it very difficult to understand why it happened and to let go
	was barely able to overcome the consequences; finds it very difficult to understand and let go, is more suspicious and vigilant
	very moved, very sad, fed up
	lives in a permanent climate of insecurity, is neglectful; never takes public transportation anymore
	yells during frequent nightmares
Severe = 3	Examples
	the aggression was a life-changing event “I am going to drag this all my life (…) it is as if my life had stopped at that moment.” Was diagnosed with PTSD and severe depression
	“my career has ended in profound sadness… I loved my job”
Clinically assessed consequences on work	
None = 0	Respondent indicates no sick leave, diminished work time, loss or leave from work as a result of the assault
Minor = 1	Sick/accident leave only (no diminished work time nor job lost/quit)
Moderate = 2	Diminished work time as a result of the assault
Severe = 3	Lost or left job as a result of the assault

The consequences were reported during the follow-up interviews. The validity of the classification in the three categories of severity is reinforced by the fact that we had sufficient information available from the qualitative data. Not only were there respondents asked about the consequences of the violent event, but how long they had lasted and to what extent the person had overcome these consequences

## Appendix 3

See Table 6.

**Table 6:** Descriptive statistics on the source population, by gender (*N* = 185)

Variables	Male population (*N* = 129)		Female population (*N* = 56)		Total population (*N* = 185)	
	*N*	%	*N*	%	*N*	%
Nationality						
Swiss	63	48.8	22	39.3	85	46
Foreign nationals	66	51.2	34	60.7	100	54
Foreigners with work/residence permit						
Yes	123	95.4	52	92.9	176	95.0
No	3	2.3	4	7.1	7	3.4
Missing	3	2.3	0		3	1.6
Occupational status						
Employee	88	68.2	46	82.1	134	72.4
Self-employed	16	12.4	4	7.2	20	10.8
Unknown	25	19.4	6	10.7	31	16.8
Sector of work						
Agriculture	1	0.8	–	–	1	0.5
Industry	13	10.1	1	1.8	14	7.6
Services	115	89.1	55	98.2	170	91.9
Generally in good health						
Yes	31	24.0	21	37.5	52	28.1
No	96	74.4	33	58.9	129	69.7
Missing	2	1.6	2	3.6	4	2.2
Previous experience of violence						
Yes	57	44.2	26	46.4	83	44.8
No	70	54.3	30	53.6	100	54.1
Missing	2	1.5	0		2	1.1

## Appendix 4

See Table 7.

**Table 7:** Descriptive statistics on the violent events (*N* = 196)

	Assaults on male victims (*N* = 137)		Assaults on female victims (*N* = 59)		Total (*N* = 196)	
	*N*	%	*N*	%	*N*	%
Type of workplace violence						
Internal	28	20.4	24	40.7	52	26.5
External	107	78.1	35	59.3	142	72.5
Internal + external	2	1.5	–	–	2	1.0
Internal violence perpetrated by						
Subordinate	3	10.0	–		3	5.5
Colleague	20	66.7	18	75.0	38	70.4
Superior	7	23.3	6	25.0	13	24.1
Time of the assault						
Day work (6 a.m.–7 p.m.)	64	46.7	36	61.0	100	51.0
Evening work (8–10 p.m.)	20	14.6	8	13.6	28	14.3
Night work (11 p.m.–5 a.m.)	50	36.5	11	18.6	61	31.1
Missing	3	2.2	4	6.8	7	3.6

## Appendix 5

See Table 8.

**Table 8:** Predictors and risk factors by categories of the severity score

Predictors (from consultation data at the time of the violent event)	Categories of severity score					
	0 = No consequences *N* = 21		1–3 = Medium level of severity *N* = 49		4+ = High severity *N* = 15	
	*N*	%	*N*	%	*N*	%
Gender						
Male	19	90.5	38	77.6	9	60
Female	2	9.5	11	22.5	6	40
Age-groups						
<35	12	57.1	14	28.6	4	26.7
35–44	6	28.6	16	32.7	4	26.7
45+	3	14.3	19	38.8	7	46.7
Initial symptoms of psychological distress						
None	14	66.7	15	28.6	3	20.0
Minor	5	23.8	15	30.6	3	20.0
Moderate	2	9.5	17	34.7	3	20.0
Severe	–	–	3	6.1	6	40.0
Initial physical wounds						
None	2	9.5	6	12.5	2	13.3
Minor	15	71.4	26	54.2	7	46.7
Moderate	4	19.1	15	31.3	6	40.0
Severe	–	–	1	2.1	–	–
Type of workplace violence						
Internal (by a coworker)	1	4.8	10	20.4	3	20.0
External (by a client, patient, etc.)	19	90.5	39	79.6	12	80.0
Both	1	4.8	–	–	–	–
Otherwise in good health						
No	4	19.1	17	35.4	6	40.0
Yes	17	81.0	31	64.6	9	60.0
Previous experience of violence (including all forms of community and family violence)						
No	9	42.9	28	57.1	9	60.0
Yes	12	57.1	21	42.9	6	40.0
Job category by awareness of violence						
Low	4	19.1	11	22.5	2	13.3
Medium	8	38.1	25	51.0	9	60.0
High	9	42.9	13	26.5	4	26.7
Was working alone						
No (one or more coworkers present)	12	57.0	21	43.8	8	53.3
Yes	9	42.9	27	56.3	7	46.7
*Risk factors (self*-*reported in follow-up interviews)*						
Perception of the employer’s response						
Adequate and helpful	14	6.7	22	45.8	3	20.0
Inadequate or nonexistent	6	29.6	17	35.4	9	60.0
No employer, self-employed	1	4.8	9	18.8	3	20.0
Perception of the response by family and friends						
Adequate and helpful	7	33.3	35	71.4	7	46.7
Inadequate or nonexistent	14	66.7	14	28.6	8	53.3
Perception of the colleagues’ response						
Adequate and helpful	11	52.4	26	54.2	6	40.0
Inadequate or nonexistent	10	47.6	16	33.3	8	53.3
No colleagues	–	–	6	12.5	1	6.7

## Footnotes

1 Patients who consulted in 2006 were not included, as this was a test year and the contents of the patients’ files were not systematized yet.

2 The term predictor was not appropriate for these variables, as they were based on data collected during follow-up interviews.

## References

[CR1] Barling J, Dupré KE, Kelloway EK (2009). Predicting workplace aggression and violence. Annu Rev Psychol.

[CR2] Bowling NA, Beehr TA (2006). Workplace harassment from the victim’s perspective: a theoretical model and meta-analysis. J Appl Psychol.

[CR3] Buckley P, Cookson H, Pakham C (2010). Violence at work: findings from the 2008/09 British Crime Survey.

[CR4] Cole LL, Grubb PL, Sauter SL, Swanson NG, Lawless P (1997). Psychosocial correlates of harassment, threats and fear of violence in the workplace. Scan J Work Environ Health.

[CR5] De Puy J, Romain-Glassey N, Gut M, Wild P, Dell’Eva A-S, Asal V (2012) Rapport final présenté à la Suva (groupe Progrès). Etude portant sur les victimes d’agressions au travail ayant consulté l’Unité de médecine des violences entre 2007 et 2010 et sur les ressources de prévention dans le canton de Vaud. Institut universitaire romand de santé au travail et Centre universitaire romand de médecine légale. Lausanne

[CR6] Dillon BL (2012). Workplace violence: impact, causes, and prevention. Work.

[CR7] European Foundation for the Improvement of Living and Working Conditions (2007) 4th European working conditions survey EWCS. Office for official publications of the european communities, Luxembourg

[CR8] European Foundation for the Improvement of Living and Working Conditions (2010) Foundation findings: physical and psychological violence at the workplace. Eurofound, Dublin

[CR9] Gates DM (2004). The epidemic of violence against health care workers. Occup Environ Med.

[CR10] Gillespie GL, Gates DM, Miller M, Howard PK (2010). Workplace violence in healthcare settings: risk factors and protective strategies. Rehabil Nurs.

[CR11] Graf M, Pekruhl U, Korn K, Krieger R, Mücke A, Zölch M (2007) Quatrième enquête européenne sur les conditions de travail en 2005. Résultats choisis du point de vue de la Suisse. SECO/Fachhochschule Nordwestschweiz, Berne et Brugg

[CR12] Hansen ÅM, Hogh A, Persson R, Karlson B, Garde AH, Ørbæk P (2006). Bullying at work, health outcomes, and physiological stress response. J Psychosom Res.

[CR13] Hogh A, Viitasara E (2005). A systematic review of longitudinal studies of nonfatal workplace violence. Eur J Work Org Psychol.

[CR14] Kowalenko T, Cunningham R, Sachs CJ, Gore R, Barata IA, Gates D, Kerr HD (2012). Workplace violence in emergency medicine: current knowledge and future directions. J Emerg Med.

[CR15] LeBlanc MM, Kelloway EK (2002). Predictors and outcomes of workplace violence and aggression. J Appl Psychol.

[CR16] Mueller S, Tschan F (2011). Consequences of client-initiated workplace violence: the role of fear and perceived prevention. J Occup Health Psychol.

[CR17] Rogers KA, Kelloway EK (1997). Violence at work: personal and organizational outcomes. J Occup Health Psychol.

[CR18] Romain-Glassey N, Ansermet C, Hofner M-C, Neuman E, Mangin P (2009). L’unité de médecine des violences: une consultation médicolégale assurée par des infirmières. Médecine et Droit.

[CR19] Schat AC, Kelloway EK (2003). Reducing the adverse consequences of workplace aggression and violence: the buffering effects of organizational support. J Occup Health Psychol.

[CR20] Sprigg CA, Martin A, Niven K, Armitage CJ (2010) Unacceptable behaviour, health and wellbeing at work. A cross-lagged longitudinal study, vol 10.1. Institution of Occupational Safety and Health (IOSH), Wigston, Leicestershire

[CR21] Tarquinio C, Duveau A, Tragno M, Fischer GN (2004). La violence au travail. Un concept à l’étude pour un état des lieux. Revue francophone du stress et du trauma.

[CR22] Taylor JL, Rew L (2011). A systematic review of the literature: workplace violence in the emergency department. J Clin Nurs.

[CR23] Wieclaw J, Agerbo E, Mortensen PB, Burr H, Tuchsen F, Bonde JP (2006). Work related violence and threats and the risk of depression and stress disorders. J Epidemiol Community Health.

[CR24] World Medical Association (2000) Helsinki Declaration of 1976, 5th Revision. World Medical Association

